# Supplementary material for: Detection of the Core Bacteria in Colostrum and Their Association with the Rectal Microbiota and with Milk Composition in Two Dairy Cow Farms
Source: Animals (Basel). 2021 Nov 24;11(12):3363. doi: 10.3390/ani11123363 (PMC8698032; doi:10.3390/ani11123363)
Supplement: Supplementary file 1 [file animals-11-03363-s001.zip › animals-1335836-supplementary.pdf]

animals

Article

Detection of the Core Bacteria in Colostrum and Their Association with the Rectal Microbiota and with Milk Composition in Two Dairy Cow Farms

Bin Chen, Guangfu Tang, Weiqing Guo, Jie Lei, Junhu Yao and Xiurong Xu *

College of Animal Science and Technology, Northwest A&F University, Xianyang 712100, China; chenbinnwafu@163.com (B.C.); tangguangfu@nwafu.edu.cn (G.T.); guoweiqing@nwafu.edu.cn (W.G.); leijie@nwafu.edu.cn (J.L.); yaojunhu2008@nwsuaf.edu.cn (J.Y.)
* Correspondence: xuxiurong@nwafu.edu.cn

Citation: Chen, B.; Tang, G.; Guo, W.; Lei, J.; Yao, J.; Xu, X. Detection of the Core Bacteria in Colostrum and Their Association with the Rectal Microbiota and with Milk Composition in Two Dairy Cow Farms. *Animals* **2021**, *11*, 3363. https://doi.org/10.3390/ani11123363

Academic Editor(s): Julia Steinhoff-Wagner and Céline Heinemann

Received: 27 July 2021
Accepted: 09 November 2021
Published: 24 November 2021

Publisher's Note: MDPI stays neutral with regard to jurisdictional claims in published maps and institutional affiliations.

Copyright: © 2021 by the authors. Licensee MDPI, Basel, Switzerland. This article is an open access article distributed under the terms and conditions of the Creative Commons Attribution (CC BY) license (http://creativecommons.org/licenses/by/4.0/).

Table S1. The core families across 100 % of the collected colostrum samples of the two farms and their average relative abundance (%).

Item	NX (*n* = 16)	NH (*n* = 18)	SEM	*p*-Value
Flavobacteriaceae	8.25	5.41	1.54	0.075
Porphyromonadaceae	0.58[b]	2.55[a]	0.67	0.006
Carnobacteriaceae	0.63	0.43	0.27	0.450
Bradyrhizobiaceae	0.62	0.74	0.29	0.663
Bacteroidaceae	0.89	1.02	0.35	0.701
Methylobacteriaceae	0.30	0.58	0.21	0.207
Lactobacillaceae	0.92	0.31	0.34	0.111
Staphylococcaceae	2.87	4.89	4.45	0.652
Pseudomonadaceae	2.25	1.17	0.43	0.160
Prevotellaceae	0.93	1.04	0.33	0.742
Sphingobacteriaceae	2.64	2.90	0.65	0.695
Intrasporangiaceae	0.46	1.12	0.36	0.064
Christensenellaceae	0.52 [b]	2.04 [a]	0.60	0.034
Enterobacteriaceae	0.89	2.35	1.37	0.296
Corynebacteriaceae	0.23 [b]	0.52[a]	0.11	0.014
Moraxellaceae	29.79 [a]	9.63 [b]	6.70	0.005
Family-XIII	0.33	0.65	0.18	0.088
Comamonadaceae	2.34	2.47	0.65	0.844
Rikenellaceae	1.99	2.04	0.72	0.944
Caulobacteraceae	0.44	0.60	0.21	0.463
Clostridiales vadinBB60group	0.31	0.24	0.10	0.473
Bacillaceae	7.44	5.45	3.82	0.606
Aerococcaceae	0.32	0.38	0.15	0.664
Rhodobacteraceae	0.55	0.82	0.18	0.139
Pasteurellaceae	0.98	0.14	0.54	0.131
Chitinophagaceae	0.44	0.46	0.10	0.856
Coriobacteriaceae	0.05[b]	0.13 [a]	0.03	0.009
Lachnospiraceae	1.71 [b]	3.13 [a]	0.59	0.022
Xanthomonadaceae	4.31[a]	2.24 [b]	0.87	0.023
Micrococcaceae	0.40	0.24	0.11	0.143
Alcaligenaceae	0.98 [a]	0.30 [b]	0.22	0.007
Sphingomonadaceae	0.69	1.01	0.23	0.175
Ruminococcaceae	5.86 [b]	10.29 [a]	2.16	0.048
Streptococcaceae	0.62	0.36	0.18	0.167

Note: [ab] Mean values in the same row with different superscripts differ ($p < 0.05$). NX, NH = colostrum samples from X dairy farm and H dairy farm.

Table S2. Part of the commensal probiotics and opportunistic pathogens presented in colostrum and their appearance frequency in the collected samples.

Part of the Probiotics	Frequency (%)	Part of the Opportunistic Pathogens	Frequency (%)
Bacillus circulans	75	*Acinetobacter lwoffii*	100
Lactobacillus	75	*Streptococcus pneumoniae*	94
Plantarum	55	*Delftia tsuruhatensis*	91
Bacillus subtilis	24	*Stenotrophomonas maltophilia*	71
Bacteroides fragilis	18	*Escherichia coli*	68
Lactobacillus casei	10	*Pseudomonas aeruginosa*	50
Ruminococcus albus	10	*Pasteurella pneumotropica*	12

Note: The sample number for this analysis is 34.

Table S3. The composition (%) and SCC number (million) in the collected colostrum.

Item	NX (*n* = 16)	NH (*n* = 18)	SEM	*p*-Value
Fat	3.17	3.63	0.58	0.425
Protein	13.35	13.90	0.69	0.414
Lactose	2.54 ^{a}	1.91 ^{b}	0.24	0.018
Total	19.62	19.19	0.90	0.879
Nitrogen	68.89 ^{b}	192.46 ^{a}	41.02	0.007
SCC	0.33	0.57	0.13	0.078

Note: ^{ab} Mean values in the same row with different superscripts differ ($p < 0.05$). NX, NH = colostrum samples from X dairy farm and H dairy farm.

Table S4. The correlation of the relative abundance of several pathogenic and beneficial bacteria with the number of SCC in colostrum.

Item	R	*p*-Value
Escherichia coli	−0.104	0.559
Pseudomonas poae	0.142	0.423
Pseudomonas putida	0.069	0.699
Pseudomonas aeruginosa	0.138	0.435
Streptococcus pneumoniae	0.139	0.432
Delftia tsuruhatensis	−0.104	0.558
Acinetobacter lwoffii	0.185	0.296
Lactobacillus plantarum	0.255	0.145
Bacillus circulans	−0.223	0.204

Note: The sample number for this analysis is 34.

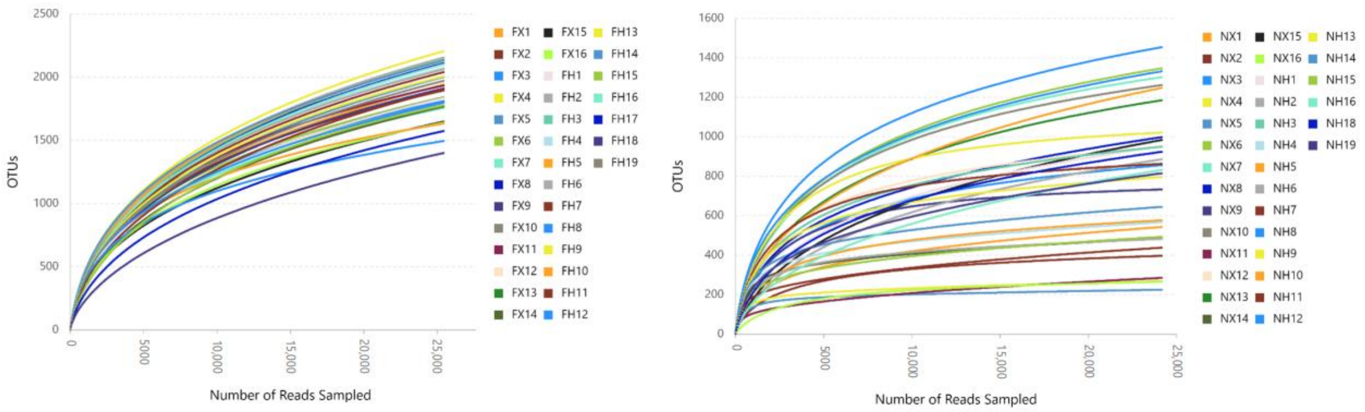

Figure S1. Rarefaction curve of all samples NX (n = 16), NH (n = 18) = colostrum samples from X dairy farm and H dairy farm, FX (n = 16), FH (n = 19) = fecal samples from X Dairy farm and H dairy farm.

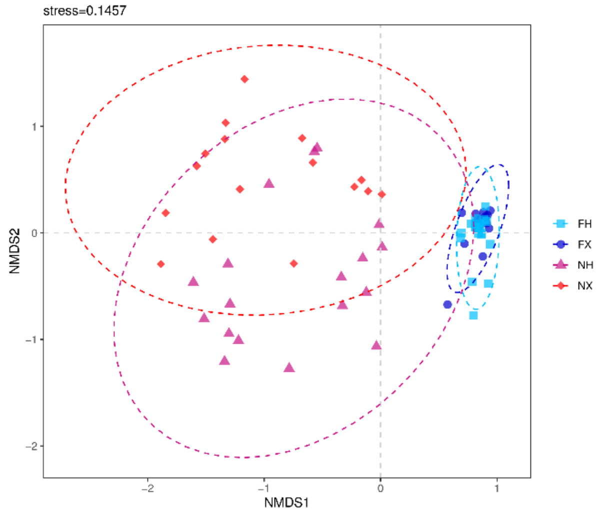

Figure S2. Non-metric multidimensional scaling of the bacterial structure of Colostrum and rectal feces samples in the two farms NX (n = 16), NH (n = 18) = colostrum samples X and Y Dairy farms; FX (n = 16), FH (n = 19) = fecal samples from X dairy farm and H dairy farm. NMDS1 characterised the dataset primarily according to the farms, whereas NMDS2 characterised the dataset primarily according to sample source (milk or feces).

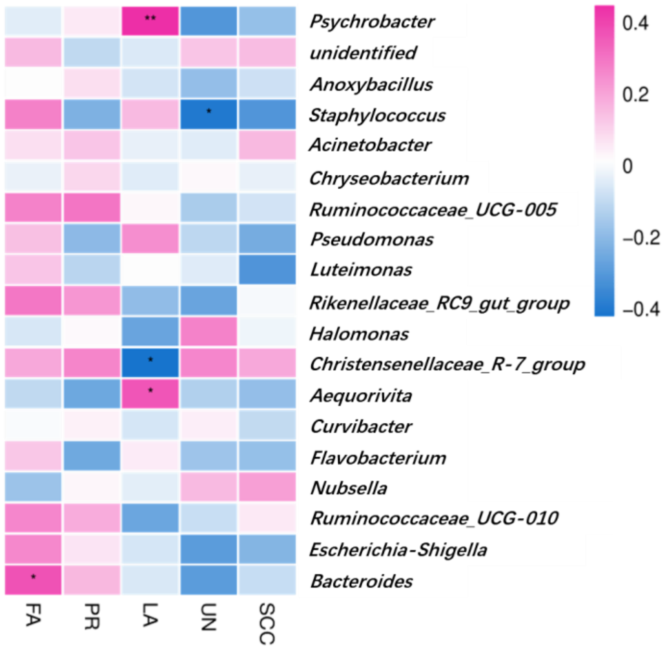

Figure S3. Correlations between colostrum composition and the 20 top genera. Note: FA = fat, PR = protein, LA = lactose, UN = urea nitrogen, SCC = somatic cell count. The sample number for this analysis is 34. * and ** meant the correlation is obvious ($p < 0.05$) and very significant ($p < 0.01$), respectively.
